# Kinase suppressor of Ras 1 and Exo70 promote fatty acid-stimulated neurotensin secretion through ERK1/2 signaling

**DOI:** 10.1371/journal.pone.0211134

**Published:** 2019-03-27

**Authors:** Stephanie Rock, Xian Li, Jun Song, Courtney M. Townsend, Heidi L. Weiss, Piotr Rychahou, Tianyan Gao, Jing Li, B. Mark Evers

**Affiliations:** 1 Department of Toxicology and Cancer Biology, University of Kentucky, Lexington, Kentucky, United States of America; 2 Lucille P. Markey Cancer Center, University of Kentucky, Lexington, Kentucky, United States of America; 3 Department of Surgery, University of Kentucky, Lexington, Kentucky, United States of America; 4 Department of Surgery, The University of Texas Medical Branch, Galveston, Texas, United States of America; 5 Department of Biostatistics, University of Kentucky, Lexington, Kentucky, United States of America; 6 Department of Molecular and Cellular Biochemistry, University of Kentucky, Lexington, Kentucky, United States of America; Medical College of Wisconsin, UNITED STATES

## Abstract

Neurotensin is a peptide hormone released from enteroendocrine cells in the small intestine in response to fat ingestion. Although the mechanisms regulating neurotensin secretion are still incompletely understood, our recent findings implicate a role for extracellular signal–regulated kinase 1 and 2 as positive regulators of free fatty acid-stimulated neurotensin secretion. Previous studies have shown that kinase suppressor of Ras 1 acts as a molecular scaffold of the Raf/MEK/extracellular signal–regulated kinase 1 and 2 kinase cascade and regulates intensity and duration of extracellular signal–regulated kinase 1 and 2 signaling. Here, we demonstrate that inhibition of kinase suppressor of Ras 1 attenuates neurotensin secretion and extracellular signal–regulated kinase 1 and 2 signaling in human endocrine cells. Conversely, we show that overexpression of kinase suppressor of Ras 1 enhances neurotensin secretion and extracellular signal–regulated kinase 1 and 2 signaling. We also show that inhibition of extracellular signal–regulated kinase 2 and exocyst complex component 70, a substrate of extracellular signal–regulated kinase 2 and mediator of secretory vesicle exocytosis, potently inhibits basal and docosahexaenoic acid-stimulated neurotensin secretion, whereas overexpression of exocyst complex component 70 enhances basal and docosahexaenoic acid-stimulated neurotensin secretion. Together, our findings demonstrate a role for kinase suppressor of Ras 1 as a positive regulator of neurotensin secretion from human endocrine cells and indicate that this effect is mediated by the extracellular signal–regulated kinase 1 and 2 signaling pathway. Moreover, we reveal a novel role for exocyst complex component 70 in regulation of neurotensin vesicle exocytosis through its interaction with the extracellular signal–regulated kinase 1 and 2 signaling pathway.

## Introduction

Obesity in the United States has reached epidemic levels, with approximately 70% of the population overweight and over 30% of overweight individuals classified as obese [1, 2]. Cancers associated with overweight and obesity account for approximately 40% of all diagnosed cancers in the United States [1]. Excess adiposity is thought to contribute to the development of cancer through the release of free fatty acids (FFAs) by adipose tissue, which have been shown to promote tumorigenesis by serving as metabolic fuel for highly proliferating cells [3–6]. FFAs have also been demonstrated to regulate expression and secretion of hormones and neuropeptides, including the gastrointestinal neuropeptide neurotensin (NT), which is implicated in the promotion of obesity and the development of several types of cancer [7–12].

NT is released in the small intestine in response to fat ingestion and has been implicated in the development of metabolic disorders and many types of cancer, including breast, pancreatic, lung, prostate, and colorectal cancer [9, 10, 13–16]. NT facilitates FFA absorption in the intestine and contributes to lipid metabolism and glucose homeostasis [12]. In mice, NT deficiency reduces intestinal fat absorption and is protective against high-fat diet-induced obesity, hepatic steatosis, and insulin resistance [12]. In humans, elevated plasma concentrations of pro-NT (a stable NT precursor fragment produced in equimolar amounts relative to NT) are associated with insulin resistance, obesity-associated metabolic disorders, and visceral adipose tissue (VAT) inflammation in obese patients [13, 17]. Moreover, high plasma pro-NT levels are predictive of the presence and severity of non-alcoholic fatty liver disease (NAFLD) [18]. Interestingly, both NT secretion and cancer growth are stimulated by unsaturated FAs [7, 19]. Pre-clinical and epidemiologic studies indicate that high intake of unsaturated fatty acids increases cancer risk and promotes cancer progression [5, 7, 19–22]. We have recently shown that NT secretion from enteroendocrine (N) cells is enhanced by common unsaturated dietary FAs, including oleic acid, palmitoleic acid, and docosahexaenoic acid (DHA) [11]. Given its intersecting role in metabolic disorders and tumorigenesis, NT may be a potential therapeutic target for metabolic diseases and cancer.

NT is secreted from N cells via the regulated secretory pathway [23]. After proteolytic processing of the NT precursor (pro-NT), active NT peptide is transported from the *trans* Golgi network and stored in secretory vesicles until NT release is triggered by extracellular stimuli [23–25]. Upon stimulation, NT-containing secretory vesicles are transported to the plasma membrane and released to the cell exterior via exocytosis [24, 25]. While the most potent extracellular stimulus regulating NT release from the small intestine is fat ingestion, the molecular mechanisms regulating FA-stimulated NT secretion are still incompletely understood [23]. We have previously demonstrated that NT gene expression is enhanced by Ras signaling and that extracellular signal–regulated kinase 1 and 2 (ERK1/2) positively regulate NT gene expression and FFA-stimulated NT peptide secretion [11, 12, 26].

ERK1/2 are mitogen activated protein kinases (MAPKs) that play a central role in the regulation of cell proliferation, differentiation, and survival [27]. ERK1/2 signaling is activated upon ligand binding to membrane receptors and subsequent activation of the small GTPase Ras, which phosphorylates and activates the MAP kinase kinase kinase (MAPKKK) Raf [28]. Activated Raf phosphorylates the MAP kinase kinases (MAPKKs) MEK1 and MEK2 (MEK1/2), which phosphorylate and activate ERK1/2 [28]. Activated ERK1/2 phosphorylates a large number of cytosolic substrates to regulate diverse cellular functions and can also be translocated to the nucleus where it activates transcription factors regulating gene expression [27–29]. Deregulation of Ras/MAPK signaling contributes to approximately one-third of human cancers [30, 31].

Coordination of the Raf/MEK/ERK protein complex and subsequent ERK1/2 phosphorylation is regulated by the scaffold protein kinase suppressor of Ras 1 (KSR1) [32–34]. KSR1 coordinates formation of the Raf/MEK/ERK signaling complex, increasing specificity of MEK phosphorylation by Ras and ERK1/2 phosphorylation by MEK [35–37]. Though a recent study presents contrasting evidence suggesting KSR1 allosterically regulates the interaction between Raf/MEK/ERK, the stimulatory effect of KSR1 on ERK1/2 remains well-substantiated [38]. KSR1 is required for Ras-induced transformation of mouse embryonic fibroblasts (MEFs) and increases proliferative potential of mammary epithelial cells when expressed at optimal levels [34]. KSR1-deficient mice exhibit reduced Ras-dependent mammary tumor formation [39, 40]. In the absence of KSR1, high molecular weight complexes containing KSR1, ERK, and MEK are abolished, and KSR1 knockout mice exhibit reduced ERK signaling [40]. Furthermore, reintroduction of KSR1 into KSR1-deficient mice rescues ERK signaling [34]. However, KSR1 is not required for ERK signaling or normal embryonic development. KSR1-deficient mice develop normally, despite attenuated ERK signaling and slightly reduced T-cell activation [35, 40]. Because KSR1 promotes Ras and ERK activation and yet is dispensable for normal development, its potential as a therapeutic target for Ras-driven cancers is being widely investigated [41]. Given the role of NT downstream of Ras and ERK1/2 signaling, we reasoned that KSR1 may also regulate NT secretion.

In this study, we examine the role of KSR1 in NT secretion and ERK1/2 signaling in the human endocrine cell lines BON and QGP-1. We demonstrate that inhibition of KSR1 reduces, while its overexpression enhances NT secretion and ERK1/2 signaling. We also show that Exo70, a component of the exocyst complex and direct substrate of ERK2, positively regulates NT secretion. These findings describe a role for KSR1 as a positive regulator of NT secretion through activation of the ERK1/2 signaling pathway and suggest that the interaction between ERK2 and Exo70 contributes to NT secretion in human endocrine cells.

## Materials and methods

### Reagents

Phospho-ERK1/2 and ERK1/2 antibodies were from Cell Signaling Technology (Danvers, MA). NT antibody for immunofluorescence was from Abcam (Cambridge, UK). Exo70 antibody for western blot analysis was from Santa Cruz Biotechnology (Dallas, TX). KSR1 antibody was from LifeSpan BioSciences (Seattle, WA). GFP antibody was from Clontech (Mountain View, CA). β-actin antibody and docosahexaenoic acid (DHA) were from Sigma-Aldrich (St. Louis, MO). PD0325901 was from Selleck Chemicals (Houston, TX). ON-TARGETplus SMARTpool (KSR1) and ON-TARGETplus Non-targeting Control Pool siRNA were from GE Dharmacon (Lafayette, CO). Non-targeting control shRNA and shRNA targeting KSR1, ERK2, and Exo70 in bacterial glycerol stock were from Sigma-Aldrich. MSCV-IRES-GFP, MSCV-KSR1-IRES-GFP, pEGFP-control and pEGFP-C3-Exo70 plasmids were from Addgene (Cambridge, MA). pECFP-N-KSR1 plasmid was from Dr. Emilia Galperin’s lab (University of Kentucky).

### Cell culture, transfection, and lentiviral transduction

The BON cell line was derived from a human pancreatic carcinoid tumor and characterized previously [42, 43]. BON cells were cultured in Dulbecco’s Modified Eagle’s Medium/Nutrient F-12 Ham supplemented with 5% fetal bovine serum (FBS) in 5% CO_2_ at 37°C. The QGP-1 cell line, derived from a pancreatic somatostatinoma (Japan Health Sciences Foundation, Osaka, Japan), was maintained in RPMI-1640 medium supplemented with 10% FBS [44]. siRNA transfections were performed using RNAiMAX (Life Technologies, Grand Island, NY). Forty-eight h after transfection, BON and QGP-1 cells were treated with 100μM DHA in serum-free medium for 90 min. Media was collected for NT-EIA and cells were lysed for western blot or RNA extraction.

For generation of cell lines expressing KSR1-targeting shRNA, BON or QGP-1 cells were plated in 6-well plates (5 × 10^5^ cells/well) in growth media containing purified non-targeting control (NTC) or KSR1-targeting shRNA lentivirus. Puromycin-selection was performed to select for cells stably expressing KSR1 shRNA and knockdown efficiency was measured via western blot or quantitative real-time PCR (qRT-PCR). For cell lines expressing ERK2 or Exo70 shRNA, lentivirus of NTC, ERK2, or Exo70 shRNAs were co-transfected with an ectopic packaging vector into 293T cells. At 48 to 72 h post-transfection, viral supernatants were collected and filtered through a 0.45-μm Surfactant Free-Cellulose Acetate sterile syringe filter. BON cells were plated in 6-well plates (5 × 10^5^ cells/well) and incubated with the viral supernatant for 24 h, followed by incubation with growth medium for an additional 24 h.

For generation of cells overexpressing KSR1 or Exo70, DNA was isolated from MSCV-IRES-GFP, MSCV-KSR1-IRES-GFP, pEGFP-control, pECFP-N-KSR1, and pEGFP-C3-Exo70 plasmids using the Qiagen Plasmid Miniprep Kit (Qiagen, Valencia, CA) according to the manufacturer’s instructions. Plasmid DNA was used to transfect BON or QGP-1 cells using Lipofectamine LTX with PLUS Reagent. Cells were collected for NT-EIA at 48–72 h post-transfection and overexpression measured via western blot or qRT-PCR.

### Immunofluorescence

BON cells were plated on glass coverslips (#1) in 24-well plates at a density of 20×10^4^/cm^2^ and transfected with pEGFP-control and pECFP-N-KSR1 plasmid DNA using Lipofectamine LTX with PLUS Reagent. 48 h after transfection, cells were fixed in 4% paraformaldehyde/PBS, permeabilized with 0.3% TritonX-100/PBS, and blocked with 0.1% bovine serum albumin/PBS. Cells were incubated with primary antibody for 1 hour, followed by Alexa Fluor-conjugated secondary antibody from Invitrogen for 30 minutes. Images were observed under a Nikon confocal microscope with 40× objective.

### Cell treatment and NT enzyme immunoassay (EIA)

Cells were plated in 24-well plates at a density of 15×10^4^/cm^2^ and grown for 48 h for DHA or drug treatment. Cells transfected with siRNA were treated with 100μM DHA in serum-free growth medium for 90 minutes. shRNA-expressing cell lines were sensitized to FFA treatment after transfection and puromycin selection and were therefore treated with 30μM DHA to reduce toxicity relative to 100μM DHA treatment. Exo70 overexpression experiments were performed with 30μM DHA for consistency with Exo70 shRNA experiments. For treatment with PD compound, cells were pretreated with PD compound for 30 minutes in serum-free growth medium and then treated with 100μM DHA plus PD for an additional 90 minutes. Media were collected for NT secretion measurement using the NT-EIA kit from Phoenix Pharmaceuticals (Belmont, CA) as described previously [45, 46] and cells were lysed for western blotting or RNA isolation. Data obtained from NT-EIA were normalized by protein concentration from corresponding cell lysates.

### Quantitative real-time PCR (qRT-PCR)

Total RNA was isolated from cells using the RNeasy Mini Kit (Qiagen, Valencia, CA) according to the manufacturer’s instructions. cDNA was synthesized from 1μg of total RNA using the High-Capacity cDNA Reverse Transcription Kit (Applied Biosystems, Foster City, CA). qRT-PCR was performed using a TaqMan Gene Expression Master Mix (#4369016) and TaqMan probes for human KSR1 (ID Hs00300134_m1) and human GAPDH (#4332649) according to manufacturer’s protocol (Applied Biosystems, Austin, TX). Relative mRNA expression was calculated using the comparative ΔΔCt method and represented as fold-change relative to internal controls.

### Protein preparation and western blotting

Protein preparation and western blotting were performed as previously described [45, 47]. Briefly, cells were lysed with lysis buffer (Cell Signaling Technology) containing 1mM phenylmethylsulfonyl fluoride (PMSF). Equal amounts of protein were resolved on 4–12% NuPAGE BisTris gels (Invitrogen, Carlsbad, CA) and electrophoretically transferred to polyvinylidene difluoride (PVDF) membranes. Membranes were incubated with primary antibodies overnight at 4°C followed by incubation with secondary antibodies conjugated with horseradish peroxidase. Membranes were developed using Amersham ECL Western Blotting Detection Reagent (GE Healthcare Life Sciences, Piscataway, NJ) or Immobilon Western Chemiluminescent HRP Substrate (Thermo Scientific, Waltham, MA). Band intensity was measured with ImageJ software using β-actin or total protein as loading controls and expressed as fold-change relative to NTCs.

### Statistical analysis

NT secretion values were normalized to protein concentration of corresponding lysates. Descriptive statistics including mean and standard deviation were calculated to summarize NT secretion. Bar graphs represent mean (± SD) fold-change of NT levels in different cell culture conditions. mRNA expression is represented in bar graphs as mean (± SD) fold-change relative to internal controls (human GAPDH). Within each experiment, comparisons across groups were accomplished using one- or two-way analysis of variance models, and pairwise comparisons were performed using contrast statements. Adjustment in p-values due to several pairwise testing within each experiment was performed using the Holm’s procedure. p-values<0.05 were considered statistically significant.

## Results

### KSR1 inhibition reduces NT secretion

**Fig 1:**
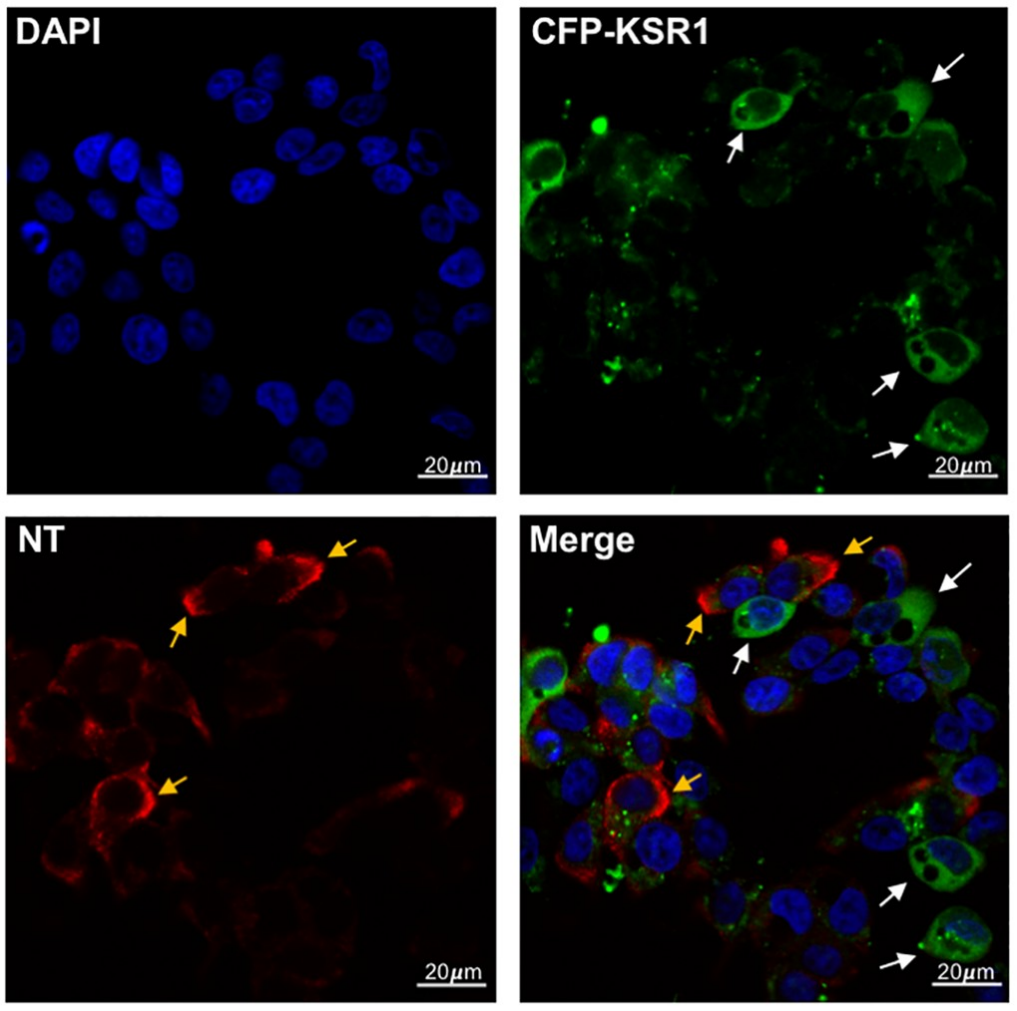
KSR1 expression stimulates NT release from BON cells. BON cells were transfected with pECFP-N-KSR1 (green) and labeled with immunofluorescent anti-NT antibody (red) and observed via confocal microscopy with 40x objective. White arrows indicate KSR1-positive cells, yellow arrows indicate NT labeling. Scale bar indicates 20μm.

KSR1 is expressed in intestinal epithelial cells, where it is protective against cytokine-induced injury [48, 49]. NT is secreted from specialized enteroendocrine (N) cells of the small intestine and is stimulated by ingestion of dietary fats [50–53]. To determine whether KSR1 is expressed in human endocrine cells and characterize its localization relative to NT, we used the BON cell line, which synthesizes and secretes NT in a manner equivalent to that of N cells in the small bowel. BON cells were transfected with CFP-tagged KSR1 plasmid DNA to enable detection of KSR1with fluorescent confocal microscopy at 488nm and subsequently labeled with fluorescent anti-NT antibody [42]. Consistent with previous evidence, we show that NT is localized to secretory vesicles in human endocrine cells, while KSR1 exhibits diffuse cytosolic staining (Fig 1). Interestingly, distribution of KSR1 and NT is largely mutually exclusive, where KSR1 expressing cells do not contain NT-containing secretory vesicles and cells retaining large amounts of NT do not express high levels of KSR1.

**Fig 2:**
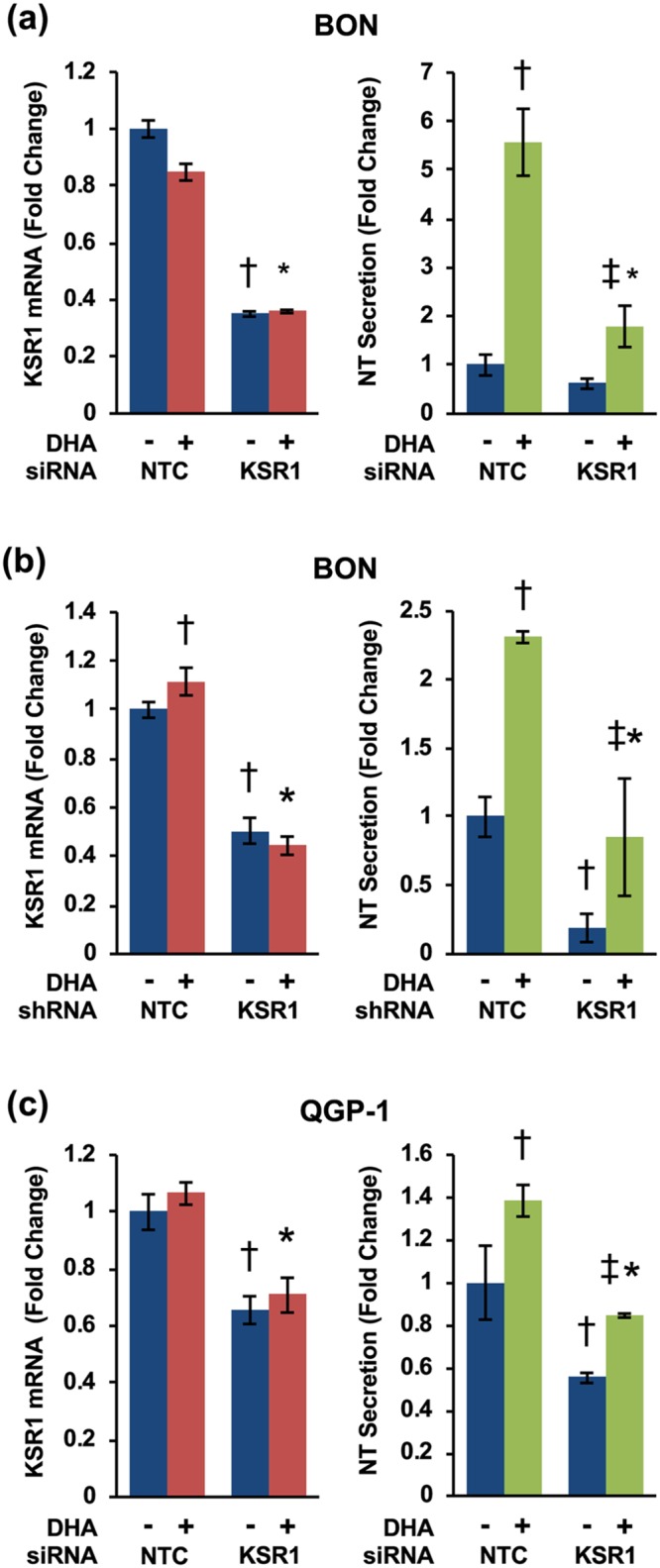
KSR1 inhibition attenuates FA-stimulated NT secretion. **(a)** BON cells were transfected with KSR1-targeting siRNA or KSR1-targeting shRNA **(b)** and treated with or without DHA for 90 min. Total RNA was isolated and qRT-PCR performed targeting KSR1 with GAPDH as the internal control (left) (†p<0.05 vs. untreated NTC; *p<0.05 vs. NTC plus DHA). Media were collected and NT-EIA performed (right) (†p<0.05 vs. untreated NTC; *p<0.05 vs. NTC plus DHA; ‡p<0.05 vs. untreated KSR1-siRNA/shRNA groups.) **(c)** QGP-1 cells were transfected with KSR1-targeting siRNA and treated with or without DHA for 90 min. Total RNA was isolated and qRT-PCR performed targeting KSR1 with GAPDH as the internal control (left) (†p<0.05 vs. untreated NTC; *p<0.05 vs. NTC plus DHA). Media were collected and NT-EIA performed (right) (†p<0.05 vs. untreated NTC; *p<0.05 vs. NTC plus DHA; ‡p<0.05 vs. untreated KSR1-siRNA group). All data represent mean +/- SD. Data are representative of three independent experiments.

We have previously demonstrated that ERK1/2 plays a stimulatory role in NT secretion [11, 54]. The function of KSR1 as a scaffold of the Raf/MEK/ERK complex has been reported to enhance ERK1/2 signaling [35–37]. To determine whether KSR1 is involved in NT secretion, we transfected BON cells with small interfering RNA (siRNA) against KSR1. Forty-eight h after transfection, cells were treated for 90 min with 100μM docosahexaenoic acid (DHA), a long-chain unsaturated FA that we have previously shown stimulates NT secretion, thereby mimicking the effects of fat-ingestion in the small bowel. KSR1 knockdown was confirmed via qRT-PCR and NT secretion was measured using NT-EIA. Consistently, inhibition of KSR1 attenuates DHA-stimulated NT secretion (Fig 2a).

To verify these results, we next established BON cell lines stably expressing KSR1-targeting shRNA. shRNA-mediated knockdown of KSR1 reduces basal and DHA-stimulated NT secretion from BON cells (Fig 2b). To further confirm these findings, we established KSR1 knockdown using siRNA in another human endocrine cell line, QGP-1. QGP-1 cells, derived from a pancreatic somatostatinoma, produce and secrete high levels of NT in response to FFA-stimulation, including DHA [11]. Consistent with the effect of KSR1 on NT secretion in BON cells, inhibition of KSR1 in QGP-1 cells reduces basal and DHA-stimulated NT-secretion (Fig 2c). Together, these data demonstrate that KSR1 regulates NT release from human endocrine cells.

### KSR1 inhibition reduces ERK1/2 phosphorylation

**Fig 3:**
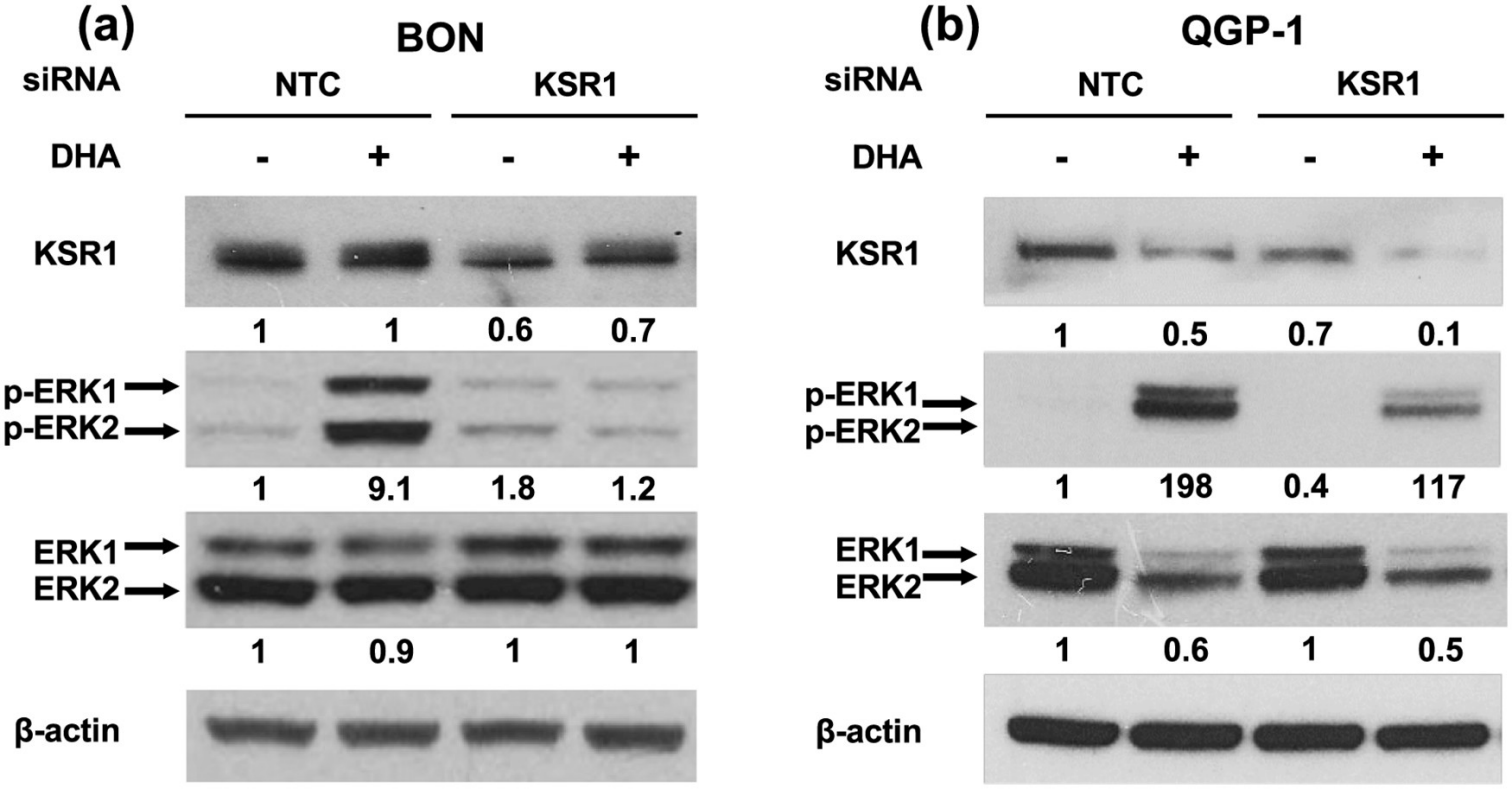
Knockdown of KSR1 attenuates ERK1/2 signaling. **(a)** BON and **(b)** QGP-1 cells were transfected with KSR1-targeting siRNA and treated with or without 100μM DHA for 90 min. Cells were lysed and western blotting analysis performed. Band intensity is indicated below the corresponding band and expressed as fold-change relative to NTC. Each blot is representative of three independent experiments.

The scaffolding function of KSR1 on the Raf/MEK/ERK complex has been demonstrated to coordinate and enhance ERK1/2 activation [32–34, 54]. KSR1-deficient mice exhibit attenuated ERK1/2 signaling and a reduction in MEK- and ERK-containing high molecular weight complexes [40]. To determine whether the effect of KSR1 on NT secretion is mediated by the ERK1/2 signaling pathway, BON and QGP-1 cells transfected with KSR1-targeting siRNA were treated with 100μM DHA to stimulate NT secretion and lysed for western blotting. Consistently, inhibition of KSR1 decreased ERK1/2 phosphorylation (p-ERK) (Fig 3). These findings are consistent with studies demonstrating that KSR1 positively regulates ERK1/2 signaling and indicate that the effect of KSR1 on NT secretion in human endocrine cells is mediated by its interaction with the Raf/MEK/ERK signaling complex.

### KSR1 overexpression stimulates NT secretion and ERK activity

**Fig 4:**
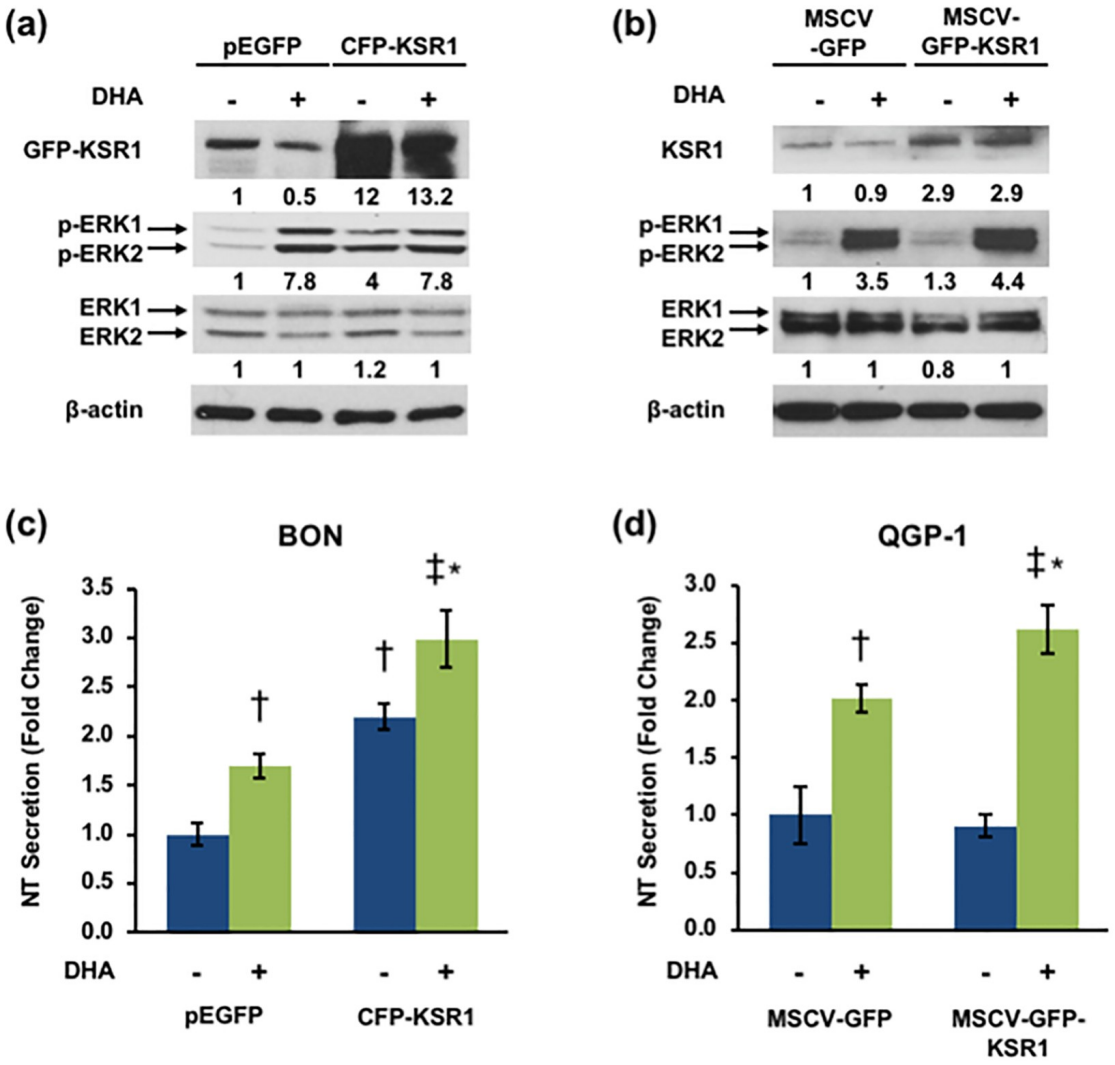
Overexpression of KSR1 enhances NT secretion and ERK activation. **(a)** BON cells were transfected with pEGFP-control and pECFP-N-KSR1 plasmid DNA and QGP-1 cells were transfected with MSCV-IRES-GFP and MSCV-KSR1-IRES-GFP plasmid DNA **(b)** and treated with or without 100μM DHA for 90 min. Cells were lysed and western blotting analysis performed. Band intensity is indicated below the corresponding band and expressed as fold-change relative to NTC. Each blot is representative of two independent experiments. **(c)** BON cells were transfected with pEGFP-control and pECFP-N-KSR1 plasmid DNA and QGP-1 cells were transfected with MSCV-IRES-GFP and MSCV-KSR1-IRES-GFP plasmid DNA **(d)** and treated with or without 100μM DHA for 90 min. Media were collected and NT-EIA performed. Panel (c): †p<0.05 vs. untreated pEGFP; *p<0.05 vs. pEGFP plus DHA; ‡p<0.05 vs. untreated CFP-KSR1; Panel (d): †p<0.05 vs. untreated MSCV-GFP; *p<0.05 vs. MSCV-GFP plus DHA; ‡p<0.05 vs. untreated MSCV-GFP-KSR1. Data represent mean +/- SD and are representative of three independent experiments.

As a scaffold protein, KSR1 can produce both inhibitory and stimulatory effects on ERK1/2 signaling when overexpressed [35]. At optimal expression levels, KSR1 facilitates maximal coordination of Raf/MEK/ERK and enhances ERK1/2 signaling, while at levels above or below this threshold, ERK1/2 signaling is inhibited. As such, the effects of KSR1 overexpression are tissue specific and based on endogenous levels of KSR1 expression [34, 35, 40]. To determine the effects of KSR1 overexpression on ERK1/2 signaling in human endocrine cells, we transfected BON cells with KSR1-overexpressing plasmids and performed western blot to assess ERK1/2 activation. Overexpression of KSR1 enhances ERK1/2 phosphorylation, suggesting that endogenous KSR1 expression in BON cells is below the threshold for coordinating maximal Raf/MEK/ERK signaling (Fig 4a and 4b). To determine whether the effect of KSR1 overexpression on ERK1/2 signaling corresponds to an increase in FFA-stimulated NT secretion, we overexpressed KSR1 in BON and QGP-1 cells and performed NT-EIA. Consistent with the observed increase in ERK1/2 signaling, DHA-stimulated NT secretion is enhanced by KSR1 overexpression (Fig 4c–4d). These data establish KSR1 as a positive regulator of FFA-mediated NT secretion in endocrine cells through activation of the Raf/MEK/ERK signaling pathway.

### Exo70 positively regulates NT secretion

**Fig 5:**
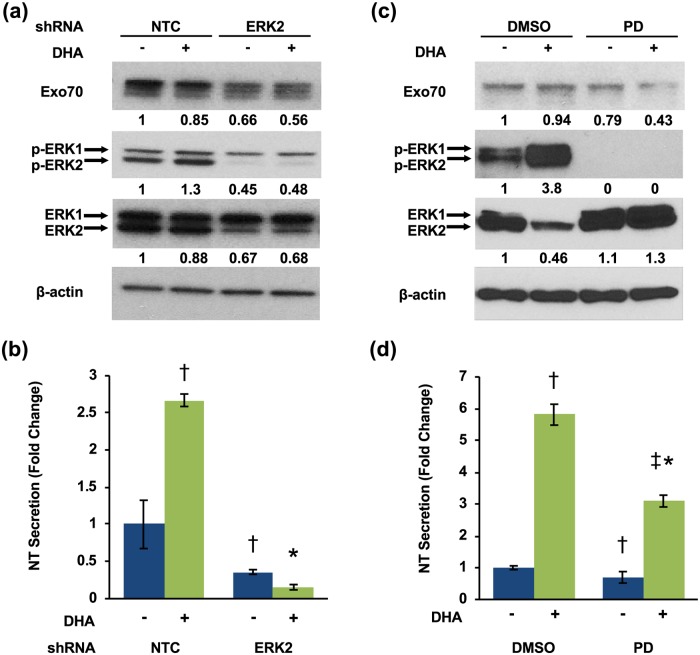
ERK1/2 inhibition attenuates Exo70 expression. **(a)** BON cells stably expressing NTC or ERK2 shRNAs were treated with or without 30μM DHA for 90 min. Cells were lysed and western blotting analysis performed. Band intensity is indicated below the corresponding band and expressed as fold-change relative to NTC. **(b)** Media were collected and NT-EIA performed (†p<0.05 vs. untreated NTC; *p<0.05 vs. NTC plus DHA; data represent mean +/- SD). **(c)** BON cells pre-treated with DMSO or PD 0325901 for 30 minutes were treated with or without 100μM DHA for 90 min. Cells were lysed and western blotting analysis performed. Band intensity is indicated below the corresponding band and expressed as fold-change relative to NTC. **(d)** Media were collected and NT-EIA performed (†p<0.05 vs. DMSO without DHA; *p<0.05 vs. DMSO plus DHA; ‡p<0.05 vs. PD without DHA; data represent mean +/- SD). All data are representative of three independent experiments.

Upon extracellular stimulation of NT release, NT-containing vesicles are transported to the plasma membrane for exocytosis and extracellular release [24, 25]. Tethering of secretory vesicles to the plasma membrane prior to secretion is mediated by the exocyst, an octameric protein complex comprised of Sec3, Sec5, Sec6, Sec8, Sec10, Sec15, Exo70, and Exo84 [55, 56]. Exo70 is a direct substrate of ERK2, which phosphorylates Exo70 at Ser250 and thereby modulates the processes mediating vesicle exocytosis [57]. Whether Exo70 regulates exocytosis of NT secretory vesicles is unknown. To determine whether Exo70 is involved in ERK-mediated NT secretion, we generated BON cell lines stably expressing ERK2 shRNA. Consistent with previous reports, ERK2 inhibition reduces Exo70 expression in BON cells (Fig 5a) [57]. NT secretion is also significantly attenuated by ERK2 knockdown (Fig 5b). To verify these results, we pre-treated BON cells with the MEK inhibitor PD 0325901 to inhibit MEK-induced phosphorylation of ERK1/2 [58]. MEK inhibition abolished ERK1/2 phosphorylation in basal and DHA-stimulated BON cells (Fig 5c) and inhibited DHA-stimulated NT secretion (Fig 5d).

**Fig 6:**
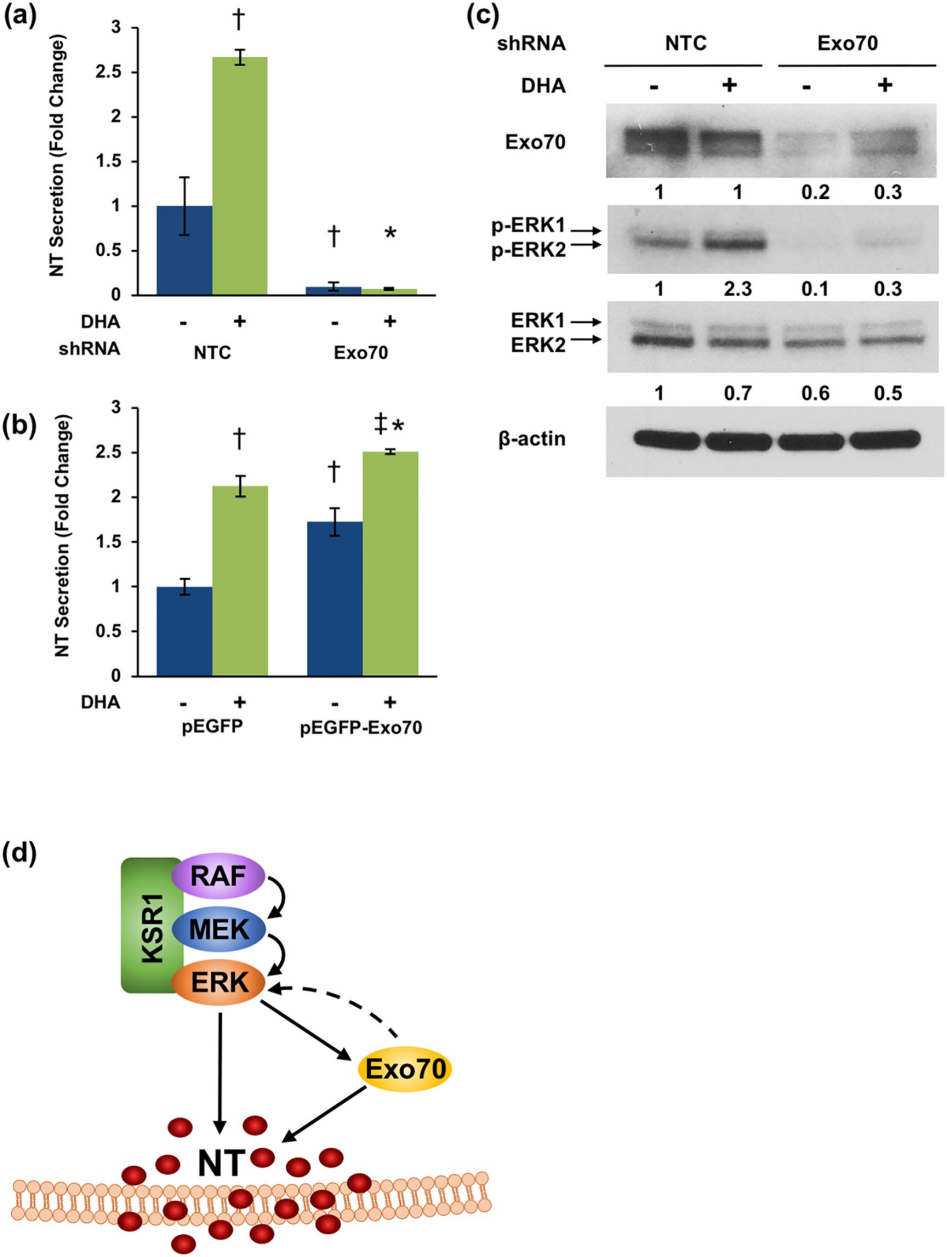
Exo70 positively regulates NT secretion. **(a)** BON cells stably expressing NTC or Exo70 shRNAs were treated with or without 30μM DHA for 90 min. Media were collected and NT-EIA performed (†p<0.05 vs. untreated NTC; *p<0.05 vs. NTC plus DHA; data represent mean +/- SD). **(b)** BON cells were transfected with pEGFP-control or pEGFP-C3-Exo70 plasmid DNA and treated with or without 30μM DHA for 90 min. Media were collected and NT-EIA performed (†p<0.05 vs. untreated pEGFP; *p<0.05 vs. pEGFP plus DHA; ‡p<0.05 vs. untreated pEGFP-Exo70; data represent mean +/- SD). **(c)** BON cells stably expressing NTC or Exo70 shRNAs were treated with or without 30μM DHA for 90 min. Cells were lysed and western blotting analysis performed. Band intensity is indicated below the corresponding band and expressed as fold-change relative to NTC. All data are representative of three independent experiments. **(d)** Proposed model of KSR1/ERK/Exo70 signaling in the control of NT secretion.

To further confirm the role of Exo70 in NT secretion, we generated BON cell lines stably expressing Exo70 shRNA and measured constitutive and FFA-stimulated NT secretion. Inhibition of Exo70 potently inhibits NT secretion from both untreated and DHA-stimulated BON cells (Fig 6a).

Conversely, overexpression of Exo70 enhances both basal and DHA-stimulated NT secretion (Fig 6b). Moreover, western blot analysis demonstrates that ERK1/2 phosphorylation is attenuated by Exo70 inhibition (6c). Collectively, these data implicate Exo70 as a positive regulator of NT secretion and suggest that the interaction between ERK1/2 and the exocyst complex may mediate exocytosis of NT vesicles from enteroendocrine cells.

## Discussion

FAs and NT play stimulatory and intersecting roles in the development of obesity and cancer. Our group recently demonstrated that stimulation of NT secretion by the unsaturated FA, DHA, is mediated by ERK1/2 signaling [11, 54]. Here, we examine the role of KSR1 on DHA-stimulated NT secretion. We show that KSR1 promotes DHA-stimulated NT secretion through the ERK1/2 signaling pathway. We also show that Exo70 inhibition reduces, while its overexpression enhances NT secretion and ERK1/2 phosphorylation. Collectively, the data presented in our current study implicate KSR1 and Exo70 as positive regulators of NT secretion that are integrated through the ERK1/2 signaling pathway.

KSR1 serves as a scaffold of the Raf/MEK/ERK complex, coordinating the spatial interactions between RAF, MEK, and ERK and enhancing specificity of their sequential phosphorylation [37, 40]. These interactions make KSR1 a potent regulator of ERK1/2 signaling [34–37]. We show that KSR1 inhibition reduces DHA-stimulated NT secretion and correspondingly inhibits ERK1/2 phosphorylation in human endocrine cells. In contrast, overexpression of KSR1 enhances NT secretion and ERK1/2 phosphorylation, and immunofluorescent labeling suggests that KSR1 expression promotes release of NT vesicles from N cells. Given the role of KSR1 as a positive regulator of ERK1/2 signaling and our previous studies showing that ERK1/2 enhances NT mRNA expression and peptide secretion, these data suggest that KSR1 positively regulates NT secretion through the Raf/MEK/ERK signaling cascade [11, 54]. KSR1 has been similarly shown to regulate secretion of insulin and inflammatory cytokines, though whether its role in secretion is predominantly positive or negative is unclear [59, 60]. KSR1-deficient mice have higher levels of basal insulin release relative to wild-type mice, suggesting that KSR1 negatively regulates insulin secretion [60]. In T-lymphocytes and splenocytes from KSR1-deficient mice, interferon gamma (IFN-γ) secretion is enhanced relative to wild-type cells, suggesting a negative role for KSR1 in IFN-γ secretion [59]. However, KSR1-deficient T cells exhibit reduced interleukin 17A (IL17A) secretion, suggesting that KSR1 enhances IL17A secretion [59].

These contrasting findings may be attributable to the role of KSR1 as a scaffold protein, which can play negative or positive roles in signal transduction depending on their expression level [35, 61–63]. Scaffold proteins facilitate maximal signaling when expressed in approximate stoichiometry with their ligands [61]. Scaffold inhibition reduces signaling due to depletion of the scaffold, yet scaffold overexpression can also inhibit signaling due to sequestration of the ligands, unless the ligands themselves are also overexpressed [61]. However, in cells endogenously expressing high levels of scaffold, inhibition may bring the scaffold and its ligands to near equivalent amounts, thereby increasing signaling [61]. The conflicting evidence regarding the role of KSR1 in secretory processes may be attributable to variation in endogenous expression levels of KSR1 and its ligands between cell types.

In mouse embryonic fibroblasts (MEFs), expression of KSR1 at 14 times endogenous levels produces maximal ERK1/2 signaling, while levels exceeding this reduce ERK1/2 signaling to levels comparable to that of KSR1-deficient cells [34]. In contrast, ectopic expression of KSR1 in 293T cells inhibits insulin- and phorbol myristate acetate (PMA)-stimulated ERK1/2 signaling [64]. We show that overexpression of KSR1 enhances ERK1/2 phosphorylation and DHA-stimulated NT secretion in neuroendocrine cells. These data suggest that, like MEFs, endogenous KSR1 expression in neuroendocrine cells is below that of the threshold for maximal ERK activation. Alternatively, components of the Raf/MEK/ERK complex may also be overexpressed in response to the downstream effects of ectopic KSR1 expression, thereby restoring stoichiometry of the complex. NT has been shown to activate ERK signaling through Ras activation [65]. Stimulation of NT by overexpression of KSR1 may therefore feedback positively on ERK1/2, optimizing signaling of the Raf/MEK/ERK complex.

As a secreted peptide, NT is subject to regulation by exocytotic processes, including vesicle trafficking, docking, and fusion with the plasma membrane [24, 25]. Exo70, a component of the exocyst complex that regulates exocytosis, is localized to lipid rafts on the plasma membrane and facilitates docking of secretory vesicles to the membrane prior to secretion [55, 56]. Exo70 is a direct substrate of ERK2, which phosphorylates Exo70 at Ser250 upon EGF stimulation [13]. We have recently demonstrated that DHA-stimulated NT secretion is mediated by an ERK1/2-dependent mechanism in neuroendocrine cells and C57/BL6 mice [11]. In our current study, we show that ERK2 knockdown reduces expression of total Exo70, though no antibodies are currently available for detection of phosphorylated Exo70. Ablation of ERK1/2 phosphorylation with the MEK inhibitor PD 0325901 also abrogated Exo70 expression and NT-secretion. These findings support other studies demonstrating that ERK2 positively regulates Exo70 and promotes its interaction with other components of the exocyst complex [57]. Activation of Exo70 by ERK1/2 signaling also mediates vesicular trafficking and matrix metalloproteinase (MMP) secretion in MDA-MB-231 human breast cancer cells [57]. Our results corroborate these findings and provide further evidence to support the role of ERK1/2 signaling as a regulator of Exo70-mediated secretory processes.

We also find that Exo70 knockdown potently inhibits both basal and DHA-stimulated NT secretion. In contrast, Exo70 overexpression enhances basal and DHA-stimulated NT secretion, suggesting a positive role for Exo70 in regulating NT secretion. Consistent with our findings, Lopez et. al [66] showed that Exo70 regulates insulin secretion. Insulin granules associate with Exo70 at the plasma membrane of β-cells, suggesting that Exo70 facilitates docking of insulin granules with the membrane [66]. Exo70 is also required for proper docking of Glut4-containing vesicles with the plasma membrane during insulin-stimulated Glut4 secretion [67]. Additionally, we show that inhibition of Exo70 attenuates ERK1/2 phosphorylation, suggesting a role for Exo70 in feedback activation of ERK1/2 signaling. Notably, Exo70 mRNA and protein is overexpressed in colon cancer, in which both ERK1/2 and NT are also heavily implicated [68]. The exocyst complex has also been implicated in the epithelial-mesenchymal transition (EMT) in mammary epithelial cells [68, 69]. Studies aimed at further delineating the interaction between Exo70 and ERK1/2 may shed light on their role in processes regulating both secretion and tumorigenesis.

In summary, the present studies demonstrate for the first time that KSR1 positively regulates FFA-stimulated NT secretion and that this effect is mediated by the stimulatory effect of KSR1 on ERK1/2 signaling. Moreover, we reveal a novel role for Exo70 in the regulation of NT secretion (Fig 6d). We also describe a role for ERK1/2 in NT secretion independent of its previously described role on NT mRNA and protein expression by demonstrating its stimulatory effect on the secretory processes mediating NT release. Collectively, these data indicate that KSR1 modulation may have therapeutic potential for diseases driven by NT gene expression and peptide secretion, such as obesity-associated metabolic disorders and Ras-driven cancers.
